# Budget Impact of Treatment-Free Remission in Treating Chronic-Phase Philadelphia-Positive Chronic Myeloid Leukemia in Lebanon

**DOI:** 10.1200/JGO.19.00012

**Published:** 2019-06-05

**Authors:** Fadia Elias, Anthony Gebran, Christina Said, Russell V. Beker, Walid Ammar

**Affiliations:** ^1^ Ministry of Public Health, Beirut, Lebanon; ^2^ Lebanese University, Beirut, Lebanon; ^3^ American University of Beirut, Beirut, Lebanon; ^4^ Russell Becker Consulting, Chicago, IL

## Abstract

**PURPOSE:**

Chronic myeloid leukemia (CML) ranks second in terms of disease-related health care expenditures at the Lebanese Ministry of Public Health (MoPH) after breast cancer. With the introduction of tyrosine kinase inhibitors (TKIs), survival of patients with CML has dramatically improved and approached that of the normal population. In recent years, several studies demonstrated that patients who achieve a deep molecular response while receiving TKI therapy could safely attempt treatment-free remission (TFR), the new treatment goal in patients with CML. The objective is to estimate the budget impact of TFR at the MoPH.

**METHODS:**

Analyses were done on 162 patients with CML receiving imatinib, nilotinib, or dasatinib, as first-line or second-line therapy, over a 4-year time horizon using MoPH drug pricing. The model assumed that patients could attempt TFR after 36 months of TKI therapy, where the last 24 months were at stable molecular response as per MoPH and National Comprehensive Cancer Network guidelines. Duration of TFR was based on European Stop Kinase Inhibitor treatment-free survival curve.

**RESULTS:**

Out of the 162 patients, 83 were eligible to attempt TFR, 36 patients were not eligible, 32 patients were lost to follow-up, two patients died as a result of CML progression, and five died as a result of other causes. The total cost of CML treatment with TFR from the time of analysis and over 4 years can be reduced by more than 7 million US dollars (57%).

**CONCLUSION:**

The model can be used to inform health care decision makers on the importance of TFR and the potential savings.

## INTRODUCTION

Chronic myeloid leukemia (CML) is a malignant disease affecting the WBCs of the human body through mutation of the *BCR-ABL* gene.^1^ Tyrosine kinase inhibitors (TKIs) that specifically target the activity of the oncogenic proteins encoded by the *BCR-ABL* gene have become the standard therapy for chronic-phase, Philadelphia-positive CML, as per international guidelines.^2,3^ TKI treatment has extensively changed the outcomes of CML by prolonging survival and increasing the number of patients achieving a deep molecular response (DMR).^4-7^

With prolonged survival on TKI therapy, CML might be added to the list of noncommunicable diseases by 2050.^8^ With the exorbitant cost of treatment per patient and per year—30,000 to 40,000 euros in Europe^9^ and approximately 31,000 US dollars ($) in Lebanon^10^—a cost-effective solution is needed.

Over the past few years, the new concept of treatment-free remission (TFR) showed promise in patients with chronic-phase CML with sustained DMR.^11,12^ TKI discontinuation has been associated with TFR rates of 50% on average.^12^ In Russia, TFR has been considered to decrease the budget burden by $14 million yearly.^13^ Although TFR is an exciting topic, careful implementation and close follow-up are needed.^14^

In Lebanon, the Ministry of Public Health (MoPH) provides cancer medication free of charge for patients who have no other insuring party.^15^ To be able to sustain its coverage, it is necessary to control the dispensing of those expensive medications. A drug scientific committee was established to review patients’ files and approve medication provision according to national cancer treatment guidelines.^10,16^ Nevertheless, the cost of cancer drugs is still a burden on the health system,^10^ which is still struggling to find its balance after the civil war and within an unstable political environment and the advent of refugees.^15,17^

The TFR concept might be one of the promising cost-saving options for the strained MoPH budget. The objective of this study was to quantify the economic impact of TFR in eligible patients with CML receiving their medication from the MoPH.

## METHODS

This is a secondary analysis of data from the MoPH Cancer Drug Scientific Committee database. Files from 162 patients with CML who had received approval for drug treatment coverage until the year 2015 were included in the analysis. The researchers analyzed de-identified data.

This is a pharmacy budget impact analysis spanning 4 years. The clinical input parameters for the simulation were based on data available at MoPH between 2012 and 2018, and other parameters were retrieved from a systematic review of the literature. The prevalence-based model was developed following the principles of good practice for Budget Impact Analysis from the International Society for Pharmacoeconomics and Outcomes Research.^18,19^ The conducted analysis was based on a third-party payer perspective. Analysis was conducted using Excel to build the model and STATA v.13 (College Station, TX) to generate population ratios.

### Patient Population

The files of 162 patients with CML receiving free treatment from the MoPH drug-dispensing center and diagnosed before 2015 were included in the analysis. This was to allow a period of at least 3 years receiving TKI therapy. New patients were not accounted for.

Eligibility criteria for TKI discontinuation therapy according to the 2018 National Comprehensive Cancer Network guidelines were applied.^3^ They include patients who were in the chronic phase of CML with no prior history of acute phase or blastic phase, patients whose duration of TKI was at least 3 years, and duration of stable molecular response (*BCR-ABL* ≤ 0.01% on the International scale) for 2 or more years. Patients with disease diagnosed before 2015 and satisfying those criteria were considered eligible to attempt TFR. Exclusion criteria comprised all patients with CML who experienced transformation into acute phase or blastic phase, those who underwent bone marrow transplantation, or those who switched lines of treatment more than twice during their treatment.

### Therapeutic Pathway

According to the MoPH guidelines, only imatinib and nilotinib are approved for first-line treatment. All TKIs are approved for second- and third-line treatment.^20^

To get approval for drug dispensing by the MoPH cancer drug scientific committee, monitoring according to the 2013 European LeukemiaNET recommendations is obligatory. Molecular and cytogenetic monitoring with a reliable polymerase chain reaction (PCR) testing that reports results on the International Scale was provided, free of charge, for all patients by a pharmaceutical company.

**FIG 1:**
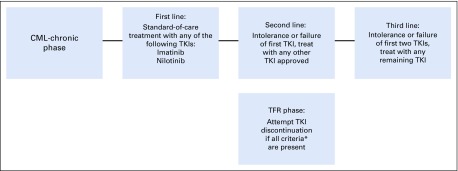
Treatment pathway considered in the analysis of patients with chronic myeloid leukemia (CML). (*) Criteria for tyrosine kinase inhibitor (TKI) discontinuation (all should be present): chronic-phase CML with no prior history of acute phase or blastic phase; duration of TKI at least 3 years; duration of stable molecular response (≤ 0.01% International Scale) for 2 or more years. TFR, treatment-free remission.

The treatment of patients with CML including the introduction of TFR follows the pathway illustrated in Figure 1.^20^ Third-line patients were not considered for TFR.

### Modeling Framework

Two scenarios were considered: a scenario without TFR where patients receive standard-of-care medication (imatinib, dasatinib, nilotinib) and another scenario with TFR where treatment discontinuation is applied. For both scenarios, both eligible and noneligible patients were included. The outcomes of the budget impact analysis model included total budget impact on MoPH in the two scenarios—with and without TFR. Model base case parameters are included in Table 1.

**TABLE 1:**
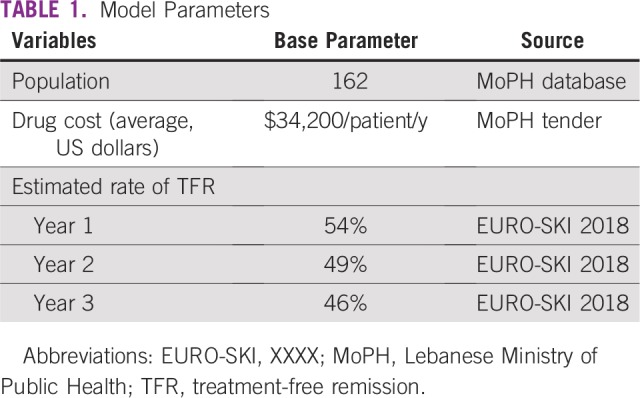
Model Parameters

### Time Horizon

The time horizon considered for the analysis is 4 years since the start of TFR in 2018 until 2022. As mentioned, eligible patients were identified by retrospective chart review. The time horizon is in line with the International Society for Pharmacoeconomics and Outcomes Research good practice guidelines for Budget Impact Analysis,^18,19^ so that the value of TFR can be assessed over many years and decision makers can foresee its impact.

### Costs Associated With Model States

In this model, we only accounted for the direct cost of treatment, which included the drug acquisition cost only. The cost of hospitalization or PCR testing was not accounted for. The average cost of the different available drugs was used. The formulas used to compute the budget impact in the two scenarios were:

1. Budget impact of scenario 1 without TFR = (EP* + NEP*) × (AYDC*)
2. Budget impact of scenario 2 with TFR
  Year 0 or Start = EP × AYDC
  Year 1 = EP × rate of TFR for year 1 × AYDC
  Year 2 = EP × rate of TFR for year 2 × AYDC
  Year 3 = EP × rate of TFR for year 3 × AYDC

where AYDC is the average yearly drug cost, EP is eligible patients, and NEP is noneligible patients.

### Assumptions

For every parameter in the model, there were assumptions. First, the rate of TFR maintenance is the same regardless of the line of treatment (eligible patients were not stratified on the basis of the line of treatment). Second, patients were compliant with their monitoring, and in case of relapse and need for treatment re-initiation, the TKI used is the same as the one previously used. Third, the cost of drugs will not change over time. Fourth, the estimated rate of TFR was based on EURO-SKI 2018.^21^ Finally, we assumed a 100% physician and patient consent rate.

## RESULTS

**FIG 2:**
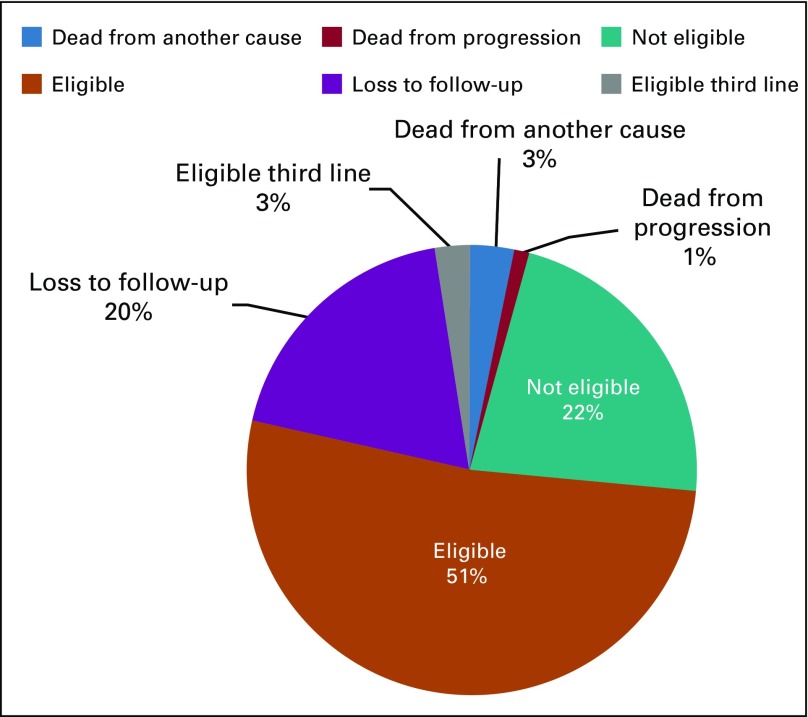
Distribution of population of patients with chronic myeloid leukemia at Lebanese Ministry of Public Health during the time of analysis.

**FIG 3:**
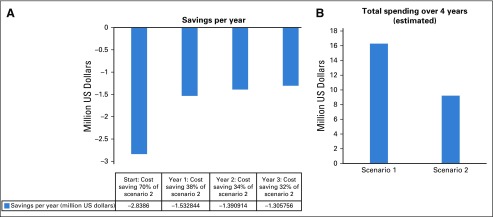
(A) Savings per year with scenario 2. (B) Estimated cost of treatment in the two scenarios over 4 years, with and without treatment-free remission over the years of analysis.

**FIG 4:**
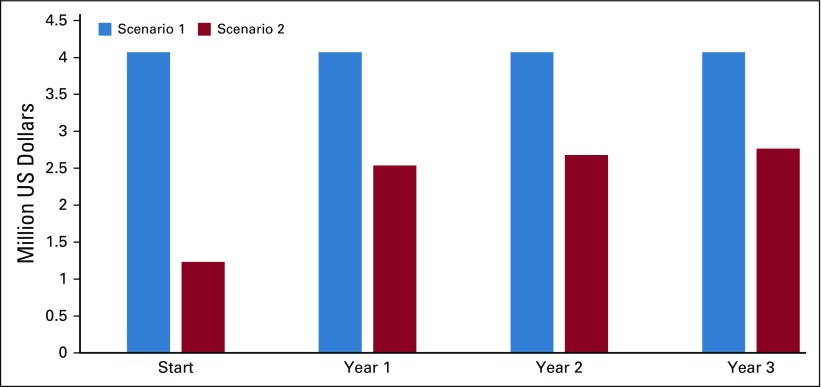
Yearly spending over 4 years.

The total number of patients with CML at MoPH having received treatment before 2015 and continued until April 2018 is 162. Eighty-three patients were receiving TKIs for more than 3 years and met eligibility criteria for TFR (Table 2). Four patients met the eligibility criteria but were not included in the analysis because they were receiving third-line treatment. The distribution of patients is illustrated in Figure 2. The main reason for loss to follow-up was patients shifting to another type of health care coverage. On the basis of the current model accounting for the different drug cost, the total estimated budget impact over 4 years without TFR is $16.3 million and with TFR is $9.2 million (Figs 3B and 4).

**TABLE 2:**
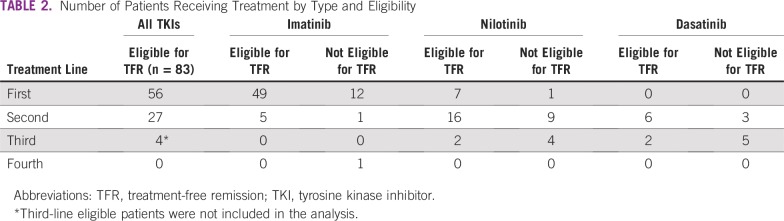
Number of Patients Receiving Treatment by Type and Eligibility

## DISCUSSION

TKIs have changed the prognosis of CML, with current life expectancy comparable to that of the general population.^4-7,22^ Recent studies showed that, after an adequate period of treatment and in the presence of deep and sustained molecular response, TKI treatment could be discontinued.^21,23-28^ Most patients with molecular relapse after interruption of TKI therapy rapidly regain response on re-initiation of treatment.^21,23-28^

CML is a costly disease. Nearly 10% of patients with CML in the United States fail to take prescribed drugs because of high costs.^29^ A recent study on the financial burden of cancer treatment in Lebanon showed that CML ranks number two in terms of disease cost, directly after breast cancer, and number one in terms of annual average drug costs per patient.^10^

Moreover, with the increasing prevalence of CML^8^ comes an increasing need for financial expenditures of the health budget, which is difficult to ensure even in the high-income countries. The MoPH covering 50% of the Lebanese population will not be able to continue providing cancer drugs for free, especially after the introduction of new expensive immunotherapy drugs, which led to an increase of 27% in MoPH cancer drug expenditures between 2014 and 2016.^29^

Effective strategies such as TKI discontinuation are needed to allow shifting from a constant accumulation of cost of treating CML to an economic plateau and stable costs.^30-32^ The savings that TFR can offer can compensate the cost of treating patients with newly diagnosed disease.

Our results show that almost half of our patients with CML are eligible to attempt TFR, which reflects the good level of management of MoPH patients with CML. It is interesting to note that we also found few third-line patients eligible for TFR. However, we did not include them in our analysis to be in line with the guidelines. The model also does not take into consideration the newly recruited patients or the newly eligible patients in TFR, which somehow compensate each other.

Similar to our finding, several studies around the world have confirmed that TFR is an effective strategy to reduce cost. One study in Russia found that implementing TFR would save $67 million over 5 years.^13^ Another retrospective analysis of 29 patients in TFR has estimated savings of around $3 million.^33^ Similarly, a study in Brazil evaluated 169 patients with CML with a median follow-up of 5 years and found that applying TFR could reduce the cost up to 95%.^34^ The updated results from the STIM1 (STop IMatinib 1) study showed that with a median follow-up of 54 months and out of 100 patients attempting TFR, the total savings were 5.5 million euros.^35^

The model confirmed the cost-saving element of TFR, allowing a budget reduction by more than half. However, there are a few assumptions in this study and several elements that should be taken into consideration for effective implementation. First, we assumed a 100% rate of physician and patient consent. In other words, TFR is a patient-physician decision. Is our medical community ready to step into the TFR era? Such a decision needs consensus between the MoPH, the physicians, and the patients.

Second, we also assumed that patients were consistent and compliant with their monitoring. This does not accurately reflect the real world. During the first 12 months, PCR should be monitored monthly, then every 6 weeks in the second 12 months, and then every 3 months afterward.^3^ This is a critical element for the success of TFR to prevent loss of response without early detection and to allow early treatment reinitiation.^3^ The TFR has been successful in clinical trials because of the tight monitoring, and additional real-world evidence is needed to confirm the integration of TFR into clinical practice.

Furthermore, the pharmaceutical companies were providing the PCR free of charge for the patients receiving treatment, and the patient was not allowed to renew his or her treatment at MoPH without the results of his or PCR. This approach allowed proper adherence and monitoring and excellent clinical outcomes. Will the pharmaceutical companies continue to provide the PCR on a monthly basis instead of quarterly during the TFR phase when the patients are off treatment? Will the patients commit to their monthly monitoring knowing that it is no longer mandatory because they are off treatment? Those are important elements to consider, knowing that loss of molecular response is silent, with no clinical symptoms until disease progression.

Third, we only took into consideration the cost of the medication. Although difficult to quantify, ideally other costs should be taken into consideration, such as the cost of adverse event management while on or off treatment (physician visits, medications, phone calls, emergency room visits, and so on), the cost of progression or death of patients who relapse without treatment re-initiation, and so on.

The model could be enhanced by taking into account all the previously mentioned factors. By doing so, the results of the study could, to a certain extent, be a better reflection of the real world.

The MoPH has been struggling for years with the increased financial burden of cancer care. Using different approaches to mitigate this increase for the sake of financial sustainability and equitable accessibility is key; however, it needs to take into consideration all the stakeholders in the health care system, including patients and physicians.

This budget impact model shows that TFR allows for a substantial reduction in CML spending estimated at more than $7 million over 4 years. This is a great tool for decision makers to maintain high levels of effectiveness and obtain a major expenditure decrease on CML treatment. In addition, it helps the sustainability of the health care coverage and supports policy makers in allocating scarce resources to more patients and other disease areas. TKI treatment discontinuation could be an effective strategy to generate additional savings at both MoPH and patients levels and to improve the patients’ quality of life; however, it needs awareness, willingness, and commitment from all the stakeholders.
